# *EGFR* Exon 18 Mutations in Advanced Non-Small Cell Lung Cancer: A Real-World Study on Diverse Treatment Patterns and Clinical Outcomes

**DOI:** 10.3389/fonc.2021.713483

**Published:** 2021-09-02

**Authors:** Haiyan Xu, Guangjian Yang, Weihua Li, Junling Li, Xuezhi Hao, Puyuan Xing, Yaning Yang, Yan Wang

**Affiliations:** ^1^Department of Comprehensive Oncology, National Cancer Center/National Clinical Research Center for Cancer/Cancer Hospital, Chinese Academy of Medical Sciences and Peking Union Medical College, Beijing, China; ^2^Department of Medical Oncology, National Cancer Center/National Clinical Research Center for Cancer/Cancer Hospital, Chinese Academy of Medical Sciences and Peking Union Medical College, Beijing, China; ^3^Department of Pathology, National Cancer Center/National Clinical Research Center for Cancer/Cancer Hospital, Chinese Academy of Medical Sciences and Peking Union Medical College, Beijing, China

**Keywords:** non-small cell lung cancer, epidermal growth factor receptor, uncommon mutation, targeted therapy, efficacy

## Abstract

**Background:**

Approximately 3–5% of patients with epidermal growth factor receptor (*EGFR*) mutation-positive non-small cell lung cancer (NSCLC) harbor exon 18 mutations. The appropriate treatment for such patients has not been clarified. The aim of this study was to investigate the response of patients with NSCLC harboring *EGFR* exon 18 mutations to different therapeutic options.

**Methods:**

Between May 2014 and September 2020, the clinical outcomes of 82 patients harboring *EGFR* exon 18 mutations who received first-generation (1G) *EGFR*-tyrosine kinase inhibitor (TKI), second-generation (2G) *EGFR*-TKI afatinib, chemotherapy, and 1G TKI in combination with chemotherapy as the initial therapy were retrospectively analyzed.

**Results:**

A total of 82 NSCLC patients harboring *EGFR* 18 mutations with whose treatment and survival outcomes were available were analyzed. The median age was 59 years, and 47 (57.3%) were female. The most common kind of *EGFR* exon 18 mutation was G719X (75.6%), followed by E709X (15.9%), E709_T710delinsD (3.6%), and other subtypes (4.9%). There was a significant difference in median progression-free survival (mPFS) by therapeutic strategy (*P* = 0.017). The mPFS of 1G TKI, 2G TKI afatinib, chemotherapy, and 1G TKI in combination with chemotherapy were 7.7 (95% CI, 4.2–11.2), 11.3 (95% CI, 5.6–17.0), 5.0 (95% CI, 2.3–17.7), and 11.1 (95% CI, 5.9–16.4) months, respectively. No significant difference in PFS was observed between afatinib and 1G TKI in combination with chemotherapy (*P* = 0.709).

**Conclusions:**

Like afatinib, 1G TKI in combination with chemotherapy might be an effective treatment option for patients harboring *EGFR* exon 18 mutations.

## Introduction

Epidermal growth factor receptor (*EGFR*) is a transmembrane glycoprotein with cytoplasmic kinase activity that can transduce essential growth factor signals from extracellular cues to cellular responses, thereby regulating cellular proliferation, differentiation, angiogenesis, and metastasis. *EGFR* mutations mainly occur in exons 18–21, which encode in the intracellular tyrosine kinase domain of *EGFR* (1). In the Asian population, the overall proportion of *EGFR* mutations was 49.1%, which is higher than that in the global population (11.9%) (2), that an in-frame deletion in exon 19 and the L858R missense mutation in exon 21 are the two most common *EGFR* mutations, which are called as the classic or sensitizing *EGFR* mutations. In contrast, 10–20% of patients with non-small cell lung cancer (NSCLC) harbor uncommon or rare *EGFR* mutation (3–6). The most prevalent of the uncommon *EGFR* mutations are point mutation and duplication in exons 18–21, *de novo* T790M mutations in exon 20, and exon 20 insertions (7). Exon 18 mutations involve missense mutations G719X and E709X, insertion-deletion (indel) mutation E709_T710delinsX, and other molecular subtypes, comprising approximately 3–5% of all the *EGFR* alterations (8, 9). Compared to patients with tyrosine kinase inhibitor (TKI)-sensitizing *EGFR* mutations, NSCLC patients with *EGFR* exon 18 mutations generally respond slightly worse to first-generation (1G) *EGFR*-TKI (7, 10, 11).

With the discovery of the oncogenic function of *EGFR*, TKIs have dramatically changed the treatment landscape of advanced NSCLC from conventional cytotoxic chemotherapy to targeted therapy in recent years. *EGFR-*TKIs are currently recognized as the first-line standard treatment for advanced NSCLC patients with *EGFR* mutations. At present, three generations of *EGFR*-TKIs include 1G reversible *EGFR*-TKIs (erlotinib, gefitinib, and icotinib), second-generation (2G) irreversible ErbB blocks (afatinib and dacomitinib), and third-generation (3G) irreversible *EGFR*-TKIs (osimertinib). Resulting from their molecular structures and biochemical differences among different *EGFR*-TKIs, their sensitivities to different *EGFR*-TKIs vary widely. A series of clinical studies have reported a response rate of 14–53.3% to 1G *EGFR*-TKIs for uncommon *EGFR* mutations, with a median progression-free survival (mPFS) of 5.98–11.6 months and a median overall survival (mOS) of 19.8–25.2 months (11–15). G719X mutations have also been demonstrated to be responsive to the 2G *EGFR*-TKI afatinib and neratinib. The overall response rates to afatinib and neratinib in NSCLC patients with G719X mutations were 77.8% and 75%, respectively, with mPFS of 13.8 months and 12.1 months and mOS of 26.9 months (9, 16), which were similar to those for classic *EGFR* mutations. At the 21^st^ World Conference on Lung Cancer (WCLC) in 2020, the results of a phase II SUMMIT basket study revealed that pretreated NSCLC patients with *EGFR* exon 18 mutations had an objective response rate (ORR) of 40% and a duration of response (DoR) of 7.5 months to neratinib, and the ORR and DoR were better than those of other *EGFR*-TKIs in previous studies (17). As neratinib was not available, on the basis of the results of the clinical trial LUX-Lung 2, LUX-Lung 3, and LUX-Lung6, involving 32 patients, the 2G *EGFR*-TKI afatinib was expanded the label by the U.S. Food and Drug Administration (FDA) in 2018 for patients with advanced NSCLC with uncommon *EGFR* mutations. In addition, a recent single-arm prospective phase II study from South Korea reported that the 3G *EGFR*-TKI osimertinib also had clinical activity in patients with uncommon *EGFR* mutations, including G719X, L861Q, and S768I, with an ORR of 53% and mPFS of 8.2 months (18).

Due to the small total sample size that exists because of the lack of randomized clinical trials and the exclusion of NSCLC patients harboring uncommon *EGFR* mutations from previous studies, the clinical outcomes of diverse treatment modalities for *EGFR-*exon- 18 mutated NSCLC have not been fully elucidated. Further study is still required to determine which treatment modality is the most effective in advanced NSCLC with uncommon *EGFR* mutations.

Therefore, we initiated a real-world study to investigate the therapeutic responses and disease progression patterns in advanced NSCLC patients harboring *EGFR* exon 18 mutations who were treated with four diverse therapy strategies: 1G *EGFR*-TKI (gefitinib, erlotinib, or icotinib), the 2G *EGFR*-TKI afatinib, chemotherapy, and a 1G *EGFR*-TKI in combination with chemotherapy.

## Materials and Methods

### Patients and Data Collection

This retrospective, single-center study was analyzed in 82 advanced NSCLC patients harboring *EGFR* exon 18 mutations who received monotherapy with a 1G- or 2G- *EGFR*-TKI, chemotherapy or a 1G *EGFR*-TKI in combination with chemotherapy in a first-line setting between May 2014 and September 2020. *EGFR* mutation testing was confirmed by amplification refractory mutation system-polymerase chain reaction (ARMS-PCR) assay or next-generation sequencing (NGS). All the gene capture panels are used in this study including 168 cancer-related NGS panel, and interrogated whole exons and critical introns for the 8 classic NSCLC oncogenic drivers, which includes *EGFR*, *KRAS*, *ALK*, *ROS1*, *BRAF*, *ERBB2*, *MET*, and *RET*. The study flow chart is presented in the **Supplementary Appendix** (**Supplementary Figure S1**).

Patients who met the following criteria were included in the analysis: age ≥18 years, Eastern Cooperative Oncology Group performance status (ECOG PS) score of 2 or less, and histologically or cytologically confirmed unresectable stage IIIB–IV or recurrent NSCLC with *EGFR* exon 18 single mutation or compound mutations. Compound mutations were defined as an exon 18 mutation in combination with another, common or uncommon, mutation in exons 18–21.

Exclusion criteria included prior treatment with concurrent chemotherapy and radiotherapy, anti-angiogenic treatment combined with *EGFR*-TKI, immunotherapy, or uncontrolled symptomatic brain metastasis.

### Treatment and Response Evaluation

*EGFR*-TKI monotherapy included the 1G TKI gefitinib (a dose of 250 mg once daily), erlotinib (a dose of 150 mg once daily), or icotinib (a dose of 125 mg three times daily), and the 2G TKI afatinib at a dose of 40 mg once daily. The chemotherapy regimens were intravenous pemetrexed (500 mg/m^2^, day 1) plus cisplatin (n = 12,75 mg/m^2^, d1), with or without anti-*VEGF* monoclonal antibody (bevacizumab 7.5 mg/kg, day 1) every 21 days as one cycle, followed by maintenance treatment with bevacizumab or pemetrexed monotherapy or a combination of bevacizumab plus pemetrexed after 6 cycles. Ten patients received carboplatin with area under the curve (AUC) equal to 5 if they were intolerable with cisplatin. Other patients received a 1G *EGFR*-TKI combined with chemotherapy every 21 days for four to six cycles, followed by maintenance treatment with pemetrexed with 1G *EGFR*-TKIs. All patients continued treatment until radiographic progression imaging examination or unacceptable toxicity as determined by their physicians.

Imaging examination at baseline was used to confirm the stage of disease, with measurable target lesions documented by computed tomography (CT) of the chest and abdomen, brain magnetic resonance imaging (MRI), or whole-body bone scans. Responses were evaluated according to the Response Evaluation Criteria in Solid Tumors (RECIST) version 1.1. The radiological images of the patients were reviewed by one radiologist, and another senior radiologist reviewed the images again in real time until an agreement was reached. Then, the imaging results were sent to hospital’s order system. Efficacy was evaluated in the first month of treatment initiation and then scanned approximately every 2 months to assess treatment response. Tumor responses were evaluated as a complete response (CR), a partial response (PR), stable disease (SD), or progressive disease (PD) for at least 6 months by the investigator according to the RECIST version 1.1.

The primary endpoint was the duration of PFS. PFS was calculated from the time of treatment initiation to the date of documented disease progression or death. The ORR was defined as the percentage of patients with confirmed CR or PR, and the disease control rate (DCR) was defined as the percentage of those with CR, PR, or SD. Overall survival (OS) was calculated from the date of first-line treatment to death or last follow-up. We recorded the pattern of first documented treatment failure according to RECIST version 1.1. Smokers were defined as current or former smokers who had smoked continuously or cumulatively in their lifetime for 6 months or more, and nonsmokers were defined as individuals who had smoked fewer than 100 cigarettes in their lifetime. All clinical data were extracted from electronic medical records in our cancer center. As an observational real-world study, it was exempted from obtaining patients’ informed consent and was approved by the institutional Ethics Review Board of National Cancer Center/Cancer Hospital, Chinese Academy of Medical Sciences, and Peking Union Medical College (approval 18-070/1648).

### Statistical Analysis

Statistical analyses were carried out by SPSS version 16.0 (SPSS Inc., Chicago, IL, USA). Patients^’^ baseline characteristics are presented as descriptive statistics. Dichotomous variables are presented as percentages and were analyzed with the chi-square test (or Fisher^’^s exact test when there was an expected frequency of less than 5). The Kaplan–Meier method with the long-rank test was used to compare PFS in different groups, which is expressed as the median value and corresponding 95% confidence index (CI). Univariate and multivariate Cox proportional hazards regression were used to evaluate predictive factors associated with PFS. A two-tailed test with *P* < 0.05 was considered statistically significant. Variables included age, sex, smoking history, clinical stage, ECOG score, histological type, molecular subtype of *EGFR* exon 18 mutation, and treatment pattern. GraphPad Prism 5.0 (GraphPad Software, San Diego, CA) was used to generate survival curves and forest plots of subtype analysis.

## Results

### Baseline Characteristics

A total of 82 patients with advanced lung adenocarcinoma harboring *EGFR* exon 18 mutations were included in the final analysis. A total of 47 (57.3%) females and 35 (42.7%) males were included, and the median age at diagnosis was 59 years old (range: 33–76 years). Forty-eight patients (58.5%) had a good ECOG PS score of 0, and 79 (96.3%) patients with lung adenocarcinoma were identified. Most patients had no smoking history (n = 57, 69.5%). Nearly a quarter of patients (n = 20) presented the central nervous system (CNS) metastasis at baseline. Sixty-nine cases (84.1%) were identified by NGS and 13(15.9%) were found by ARMS-PCR assay. All specimens were available for genetic testing *via* tissue biopsy (n = 77) or peripheral blood samples (n = 5). Of them, tissue samples originated from the lung and pleural effusion (n = 63), lymph nodes (n = 9), and other sites (n = 5). The baseline characteristics of patients were well balanced among the different treatment arms (in **Table 1**).

**Table 1:** Baseline characteristics of patients.

Baseline Characteristics	1G TKI (n = 24)	2G TKI (n = 21)	CT (n = 22)	1G TKI + CT (n = 15)	*P*
Age					0.428
≤60	12 (50.0)	8 (38.1)	14 (63.6)	6 (40.0)	
>60	12 (50.0)	13 (61.9)	8 (36.4)	9 (60.0)	
Sex					0.412
Female	15 (62.5)	14 (66.7)	12 (54.5)	6 (40.0)	
Male	9 (37.5)	7 (33.3)	10 (45.5)	9 (60.0)	
Smoking					0.136
Yes	4 (16.7)	5 (23.8)	9 (40.9)	7 (46.7)	
No	20 (83.3)	16 (76.2)	13 (59.1)	8 (53.3)	
ECOG score					0.265
0	13 (54.2)	16 (76.2)	12 (54.5)	7 (46.7)	
1–2	11 (45.8)	5 (23.8)	10 (45.5)	8 (53.3)	
Histological types					0.622
ADC	22 (91.7)	21 (100)	21 (95.5)	15 (100)	
Non-ADC	2 (8.3)	0 (0)	1 (4.5)	0 (0)	
Tumor stage					0.569
IIIb	1 (4.2)	3 (14.3)	3 (13.6)	1 (6.7)	
IV	23 (95.8)	18 (85.7)	19 (86.4)	14 (93.3)	
Brain metastases					0.139
Yes	9 (37.5)	6 (28.6)	4 (18.2)	1 (6.7)	
No	15 (62.6)	15 (71.4)	18 (81.8)	14 (93.3)	
Molecular subtype					0.289
G719X	20 (83.3)	16 (76.1)	12 (54.6)	14 (93.3)	
E709X	3 (12.5)	3 (14.3)	6 (27.3)	1 (6.7)	
E709_T710 delinsD	1 (4.2)	1 (4.8)	1 (4.5)	0 (0)	
G724S	0 (0)	1 (4.8)	3 (13.6)	0 (0)	

1G, first-generation; 2G, second-generation; TKI, tyrosine kinase inhibitors; CT, chemotherapy; ECOG, Eastern Cooperative Oncology Group; ADC, adenocarcinoma; EGFR, epidermal growth factor receptor.

### Subtypes of *EGFR* Exon 18 Mutations Among All Patients With or Without CNS Metastasis or Coexisting Genetic Alterations

**Figure 1:**
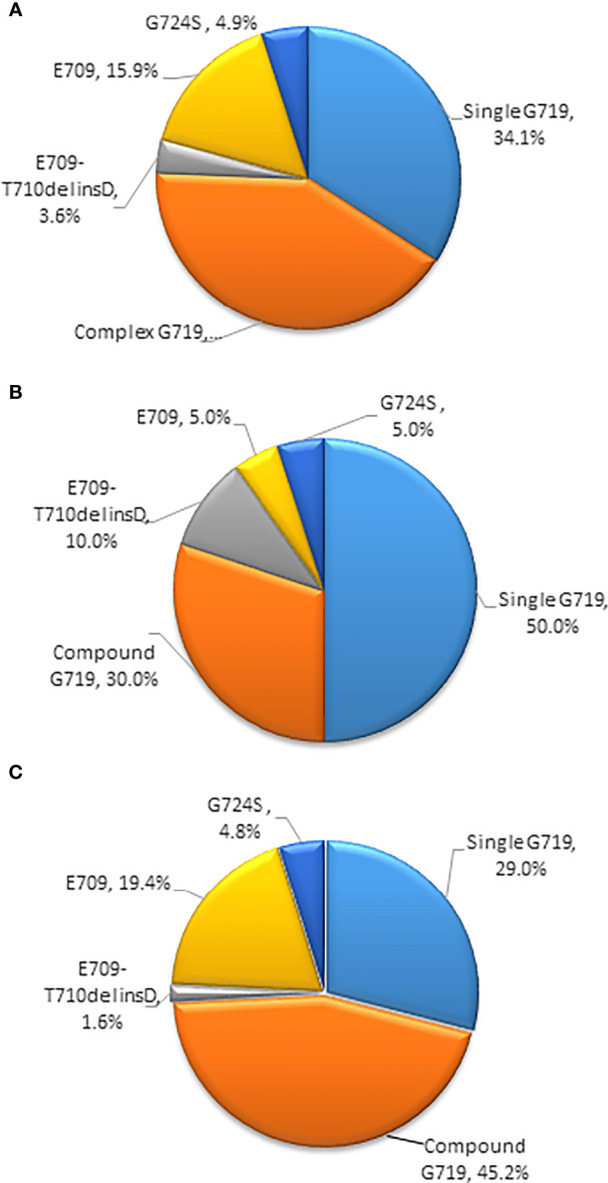
**(A)** Percentages of subtypes of *EGFR* exon 18 mutations among all patients **(B)** Percentages of subtypes of *EGFR* exon 18 mutations with brain metastases **(C)** Percentages of subtypes of *EGFR* exon 18 mutations without brain metastases. (*EGFR*, epidermal growth factor receptor).

Among the 82 patients with *EGFR* exon 18 mutations, the most common *EGFR* exon 18 mutation was G719X (n = 62, 75.6%), followed by E709X (n = 13, 15.9%), E709_T710delinsD (n = 3, 3.6%), and G724S (n = 4, 4.9%) (**Figure 1**). G719X substitutions G719A, G719C, and G719S and unknown subtypes were found in 62 patients, among them 34.1% harbored single G719X mutation and 41.5% harbored a compound G719X mutation. Thirteen patients carried E709X mutation. Nine out of 13 patients harbored E709K with G719A/C/S as well, and the other 4 patients carried E709K/A/Q with L858R. The detailed molecular subtypes of *EGFR* exon 18 mutations were shown in **Table 2**.

**Table 2:** Different subtypes of *EGFR* exon 18 mutation found in this sample.

Subtypes of *EGFR* exon 18 mutation	Number (n = 82, %)
G719X	62 (75.6)
G719A	15
G719C	1
G719S	3
G719 unknown	9
G719A + S768I/S720F/L861R/R766	3/1/1/1
G719C + S768I/L861Q/L861R/K714N	11/1/1/1
G719S + S768I/L858R	7/3
G719 unknown + S768I	4
E709X	13 (15.9)
E709K + G719A/C/S	3/2/4
E709K/A/Q + L858R	4
E709_T710delinsD	3 (3.6)
G724S + *EGFR*19/S768I	3/1 (4.9)
Total	82 (100)

EGFR, epidermal growth factor receptor.

Among the 20 patients who presented with baseline CNS metastasis at the time of the primary diagnosis, the most common *EGFR* exon 18 mutation subtypes were G719X (n = 16, 80%) and E709_T710delinsD (n = 2, 10%). Other molecular subtypes were E709X and G724S mutations (n = 2, 10%). With a limited number of cases, no specific *EGFR* exon 18 subtype had an increased tendency for CNS metastasis at diagnosis **(** **Figure 1** **)**.

Sixty-nine cases were tested by NGS, and 10 (12.2%) had coexisting genetic alterations. *TP53* mutation was detected in four cases, and *EGFR* amplification was detected in two samples. In addition, *MET* amplification and mutations of *PTEN*, *FGFR3*, and *HER2* were detected in one case each.

### Therapeutic Responses and Survival Analysis After First-Line Therapy in Patients With *EGFR* Exon 18 Mutations

At the time of the cutoff date (December 31, 2020), the median follow-up time since the diagnosis of advanced or metastatic disease was 30.8 months (range: 1.8–81.0 months). Of the 82 patients, 1G *EGFR*-TKI was administered in 24 patients, another 21 patients received afatinib, 22 patients received chemotherapy, and 15 patients received a 1G *EGFR*-TKI combined with chemotherapy. The response rates to 1G *EGFR*-TKI, 2G *EGFR*-TKI afatinib, chemotherapy, and 1G *EGFR*-TKI combined with chemotherapy were 25.0%, 52.4%, 40.9%, and 46.7% (*P* = 0.276), and the DCRs were 78.2%, 76.1%, 47.8%, and 86.7%, respectively (*P* = 0.021).

**Figure 2:**
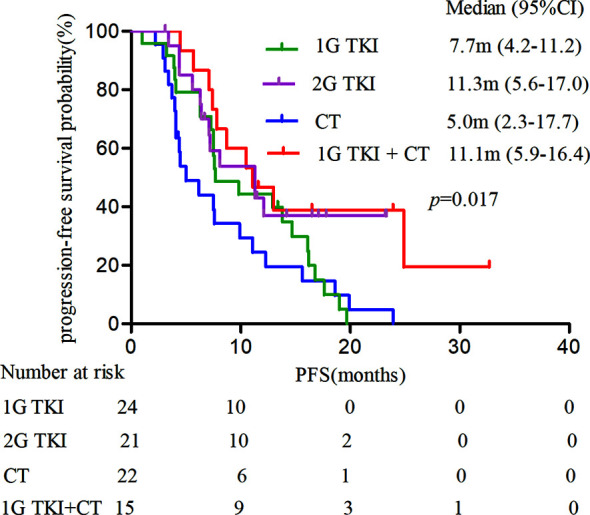
Kaplan-Meier curves of PFS in patients harboring *EGFR* exon 18 mutations treated with different treatment modalities. (PFS, progression-free survival).

Most patients harboring *EGFR* exon 18 mutations receive *EGFR*-TKI treatment involving a 1G, 2G, or 3G TKI in the first-line setting, though these make up a small sample in all. A summary of the different therapeutic strategies for *EGFR* exon 18 mutations from various studies is shown in **Table 3**. In our study, the 2G TKI afatinib had relatively long PFS for NSCLC patients with *EGFR* exon 18 mutations. The mPFS differed significantly between patients treated with different treatment strategies (*P* = 0.017). The mPFS of 1G *EGFR*-TKI, the 2G EGFR-TKI afatinib, chemotherapy, and 1G *EGFR*-TKI in combination with chemotherapy were 7.7 (95% CI, 4.2–11.2), 11.3 (95% CI, 5.6–17.0), 5.0 (95% CI, 2.3–17.7), and 11.1months (95% CI, 5.9–16.4), respectively **(** **Figure 2** **)**.

**Table 3:** Comparisons of different treatments for *EGFR* exon 18 mutations from various studies.

Study	N	*EGFR* exon 18 subtype	Treatment	ORR	PFS
This study	82	G719X	A	52.4%	11.3 (5.6–17.0)
		E709X	G/E/I + CT	46.7%	11.1 (5.9–16.4)
		DelE709_T710ins D	G/E/I	26.1%	7.7 (4.3–11.1)
		Complex G724S	CT	39.1%	6.2 (1.8–10.6)
Passaro et al. (19)	42	Single 18 mutation	G/E/A	31.0%	8.3 (4.9–11.7)
Zhang et al. (13)	22	Single G719X	G/E/I	22.7%	7.6 (4.9–10.4)
		Complex G719 mutations			
Chui et al. (14)	78	Single G719X mutation	G/E	36.8%	6.3
	9	G719X + L861Q		88.9%	NR
	10	G719X + S768I		50.0%	NR
Yang et al. (7)	8	SingleG719X	A	77.8%	13.8 (6.8–NE)
	10	Complex G719 mutation			
Cho et al. (18)	19	G719X	O	53.0%	8.2

A, afatinib; G, gefitinib; E, erlotinib; I, icotinib; O, osimertinib; CT, chemotherapy; NR, not reached.

We further analyzed the clinical outcomes in advanced NSCLC patients harboring *EGFR* exon 18 mutations treated with four diverse therapeutic strategies. Compared to the 2G *EGFR*-TKI afatinib as the standard of care, a significant survival drop was observed with chemotherapy alone (HR = 2.261, 95% CI: 1.108–4.611, *P* = 0.025), while there was no significant difference in PFS between 1G *EGFR*-TKI or 1G *EGFR*-TKI combined with chemotherapy and afatinib (HR = 1.632, 95% CI: 0.806–3.304, *P* = 0.173 and HR = 0.774, 95% CI: 0.323–1.854, *P* = 0.566, respectively), and the mOS was not reached in any subgroup.

### Univariate and Multivariate Analyses for PFS by the Cox Regression Model

Univariate analysis showed that the PFS of advanced NSCLC patients with *EGFR* exon 18 mutations was significantly associated with molecular subtype (*P* < 0.001) and treatment pattern (*P* = 0.023) (**Table 4**). Although there was no statistically significant difference in PFS between those with and without brain metastasis due to the small sample size, the status of brain metastasis might have affected the outcome of PFS in a previous study (20). Therefore, the status of brain metastasis, molecular subtype, and treatment pattern was entered into the multivariate Cox regression model. Multivariate analyses confirmed that *EGFR* exon 18 molecular subtype and treatment pattern were independent predictors of PFS in advanced NSCLC patients with *EGFR* exon 18 (*P* < 0.05, **Table 5**).

**Table 4:** Univariate survival analyses for PFS.

Variable	B	SE	HR	95% CI	*P*
Age (≥60 *vs.* <60)	-0.064	0.250	0.938	0.575–1.530	0.798
Sex (male *vs.* female)	0.507	0.265	1.061	0.988–2.792	0.056
Smoking history (yes *vs.* no)	0.209	0.276	0.450	0.717–2.117	0.450
Histological types (ADC *vs.* Non-ADC)	-0.043	0.595	0.958	0.299–3.074	0.943
Clinical stage (IIIb *vs.* IV)	-0.023	0.431	0.978	0.420–2.275	0.958
ECOG score (0 *vs.* 1–2 points)	-0.034	0.254	0.966	0.587–1.590	0.893
Brain metastases at baseline (yes *vs.* no)	0.489	0.296	1.631	0.913–2.913	0.099
Molecular subtype					<0.001
(E709-T710delinsD *vs.* G719X)	0.865	0.730	2.376	0.568–9.937	0.236
(E709X *vs.* G719X)	-0.619	0.384	0.539	0.254–1.144	0.107
(G724S *vs.* G719X)	2.675	0.623	14.515	4.277–49.259	<0.001
Treatment patterns					0.023
(CT *vs.* 2G TKI)	0.816	0.364	2.261	1.108–4.611	0.025
(1G TKI *vs.* 2G TKI)	0.490	0.360	1.632	0.806–3.304	0.173
(1G TKI+CT *vs.* 2G TKI)	-0.256	0.445	0.774	0.323–1.854	0.566

HR, hazard ratio; CI, confidence interval; ADC, adenocarcinoma; ECOG, Eastern Cooperative Oncology Group; TKI, tyrosine kinase inhibitor; CT, chemotherapy; 1G, first-generation; 2G, second-generation.

**Table 5:** Predictors of PFS analyzed by multivariate Cox regression.

Variable	B	SE	HR	95% CI	*P*
Brain metastases at baseline (yes *vs.* no)	0.130	0.341	1.138	0.583–2.221	0.704
Molecular subtype					<0.001
(E709-T710delinsD *vs.* G719X)	0.864	0.804	2.372	0.491–11.460	0.283
(E709X *vs.* G719X)	-0.802	0.403	0.448	0.204–0.987	0.046
(G724S *vs.* G719X)	2.219	0.659	9.199	2.528–33.470	0.001
Treatment patterns					0.023
(CT *vs.* 2G TKI)	0.874	0.382	2.398	1.135–5.066	0.022
(1G TKI *vs.* 2G TKI)	0.479	0.381	1.614	0.765–3.406	0.209
(1G TKI + CT *vs.* 2G TKI)	-0.245	0.459	0.783	0.318–1.926	0.594

HR, hazard ratio; CI, confidence interval; TKI, tyrosine kinase inhibitors; CT, chemotherapy; 1G, first-generation; 2G, second-generation.

### Subgroup Analysis by Different Treatment Pattern

**Figure 3:**
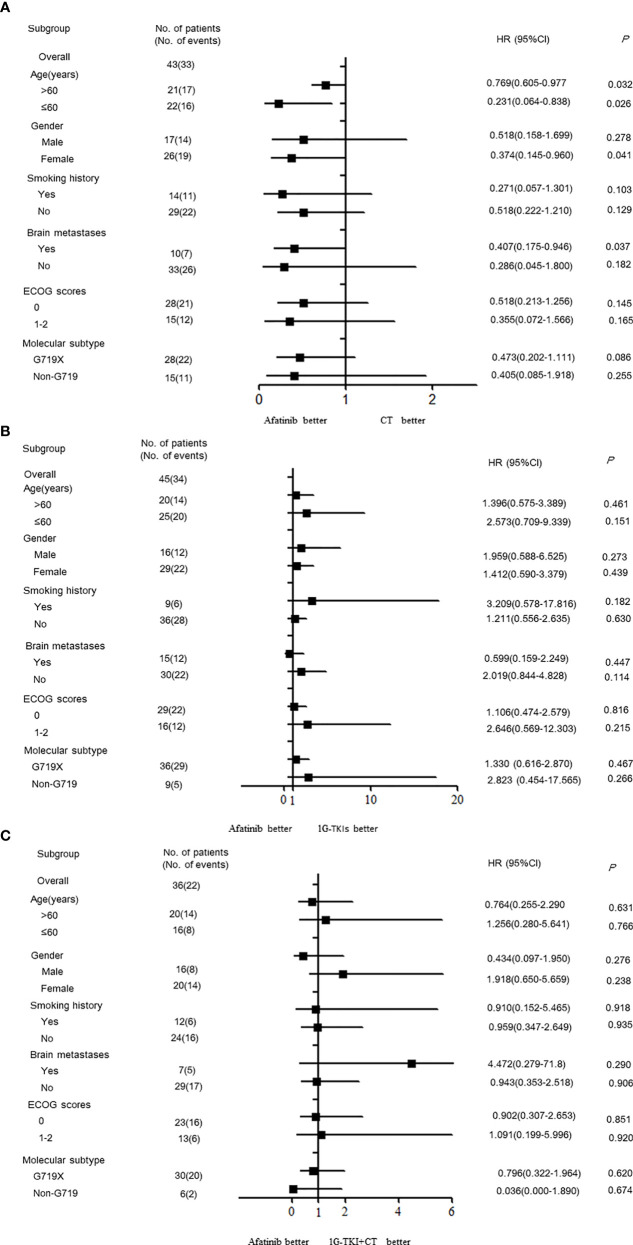
Forest plots by the various demographics and disease characteristics. **(A)** afatinib *vs.* CT **(B)** afatinib *vs.* 1GTKI **(C)** afatinib *vs.* 1G TKI+CT. (CT, chemotherapy; 1G-TKI, first-generation tyrosine kinase inhibitor).

Subgroup analyses of PFS based on investigator assessment are presented according to baseline characteristics. The PFS of patients in the afatinib cohort was significantly better than that in the chemotherapy cohort among females and among patients without brain metastases, regardless of age (*P* < 0.05, **Figure 3A**). In contrast, no significant PFS benefit was observed between the afatinib cohort and the 1G TKI or in the 1G TKI in combination with chemotherapy cohort in any subgroup, including the age, sex, smoking history, ECOG score, brain metastasis, and molecular type subgroups (*P* > 0.05, **Figures 3B, C**).

### Disease Progression Patterns in Patients With *EGFR* Exon 18 Mutations

At the cutoff date, 65 (79.3%) of 82 patients presented disease progression. Intrathoracic metastases were the most common progressive pattern in patients harboring *EGFR* exon 18 mutations, accounting for 55.3% (n = 36), followed by 24.6% (n = 16) in the brain and 20.1% (n = 13) in other organs, including the liver, bone, adrenal, and lymph nodes, among all patients.

Different treatment modalities had a tendency to develop distinct progressive patterns (*P* = 0.037). Intrathoracic metastases were observed in 26.1% of progressive patients treated with chemotherapy, 12.3% of those treated with 1G *EGFR*-TKI, 9.2% of those treated with 1G *EGF*R-TKI in combination with chemotherapy, and only 7.7% of those treated with afatinib. The proportion of brain metastases was the highest in patients treated with 1G *EGFR*-TKIs (n = 10, 15.4% of progressive patients). The rates of brain metastases in patients treated with afatinib, chemotherapy, and 1G *EGFR*-TKI in combination with chemotherapy were 4.5% (n = 3), 3.0% (n = 2), and 1.5% (n = 1), respectively. The disease progression patterns are listed in the **Supplementary Appendix** (**Supplementary Figure S2**).

## Discussions

With the development of comprehensive molecular profiling of NSCLC, an increasing number of uncommon *EGFR* mutations have been revealed other than the known *EGFR*-sensitive mutations, including *EGFR* exon 19 deletion and the exon 21 L858R missense mutation. *EGFR* exon 18 mutations are listed as uncommon mutations involving missense and deletion/insertion mutations. However, patients with *EGFR* exon 18 mutations have heterogeneous outcomes after taking different *EGFR*-TKIs (9, 19, 21). To date, this is the largest of treatment outcome reported in NSCLC patients harboring *EGFR* exon 18 mutation. In this study, we analyzed the distribution of subtypes of *EGFR* exon 18 mutation and the clinical outcomes of advanced NSCLC patients with *EGFR* exon 18 mutations receiving different treatment strategies as first-line therapy. In line with previous studies (22, 23), the most common *EGFR* exon 18 mutation was G719X (75.6%), followed by E709X (15.9%), E709_T710delinsD (3.6%), and other subtypes (4.9%). Single G719X mutation involved different subtypes (G719A, C, D, S, and V), the most common of which was G719A (n = 15, 53.6%). A total of 41.5% of *EGFR* G719X mutations were identified as part of compound mutations (n = 34), and approximately one-third of G719X mutations presented in combination with the S768I mutation.

Given the low prevalence of *EGFR* exon 18 mutations and the lack of prospective head-to-head research data, NSCLC patients harbor uncommon *EGFR* exon 18 mutations. The results of different treatment options are still relatively sparse. Our study provides real-world therapeutic responses in advanced NSCLC patients with *EGFR* exon 18 mutation treated with different treatment strategies in the first-line setting. Patients treated with a 2G *EGFR*-TKI (afatinib) or a 1G *EGFR*-TKI in combination with chemotherapy had a relatively better response rate than those treated with a 1G *EGFR*-TKI or chemotherapy alone. The response rates to 1G *EGFR*-TKI, afatinib, chemotherapy, and 1G *EGFR*-TKI combined with chemotherapy were 25.0%, 52.4%, 40.9%, and 46.7% (*P* = 0.276), and the DCRs were 78.2%,76.1%, 47.8%, and 86.7%, respectively (*P* = 0.021).

We also analyzed the clinical outcomes in advanced NSCLC patients harboring *EGFR* exon 18 mutations treated with the four different treatment strategies. The mPFS of patients treated with different treatment strategies was significantly different (*P* = 0.017). Taking the 2G *EGFR*-TKI afatinib as the standard of care, a significant deficit in PFS was observed in patients who had chemotherapy alone (*P* = 0.023): The mPFS of patients treated with afatinib was 11.3 months, compared with 5.5 months in those treated with chemotherapy. There was no significant difference in PFS between the 1G *EGFR*-TKI group or the 1G *EGFR*-TKI combined with chemotherapy group and afatinib (*P* > 0.05). The mPFS of the 1G *EGFR-*TKI group and the 1G *EGFR-*TKI in combination with chemotherapy group was 7.7 months and 11.1 months, respectively. Furthermore, multivariate analyses demonstrated that treatment pattern was an independent predictor of PFS in NSCLC patients with *EGFR* exon 18 mutations. Similarly, Kobayashi et al. (24) reported that patients with specific exon 18 mutations were more sensitive to 2G *EGFR*-TKI than 1G *EGFR*-TKI or the 3G *EGFR*-TKI osimertinib compared with *EGFR* exon 19 deletion patients, and to some degree, they generally tended to be resistant to gefitinib or erlotinib. An *in vitro* study in cell lines also showed a better response to afatinib and neratinib than to gefitinib and erlotinib, with respective IC90s of 0.9 nM and 1.1 nM, in cell with G719A (25).

Based on the LUX-Lung clinical trials, afatinib was approved for the treatment of metastatic NSCLC harboring *EGFR* S768I, L861Q, and/or G719X by the U.S. FDA in 2018. However, afatinib cannot cover all uncommon *EGFR* mutations and severe dose-limiting toxicities were observed in these trials, so those patients should explore alternative treatment strategies. A phase III clinical trial (NEJ009) (26) demonstrated that gefitinib in combination with pemetrexed and carboplatin improved PFS and OS compared with *EGFR*-TKIs alone for untreated advanced NSCLC with classic *EGFR* mutations. However, the efficacy of gefitinib in combination with pemetrexed and carboplatin in uncommon *EGFR* mutations is unknown. Our study confirms the results of NEJ009, indicating that 1G in combination with chemotherapy has a good PFS outcome for patients not only with common *EGFR* mutations but also with uncommon *EGFR* exon 18 mutations. Compared to afatinib treatment as the standard of care, there was a similar survival time in PFS of 1G *EGFR*-TKI in combination with chemotherapy (*P* = 0.709), which indicated that 1G *EGFR*-TKI in combination with chemotherapy might be a potentially effective option for the treatment of NSCLC patients with *EGFR* exon 18 mutations. It was of note that a subgroup included in the present analysis had received 1G *EGFR*-TKI plus chemotherapy, which enriched the treatment data for uncommon *EGFR* mutation. In addition to treatment strategy, molecular subtype was associated with PFS in our NSCLC patients with *EGFR* exon 18 mutation. Therefore, comprehensive and detailed molecular subtype testing is of great importance to clinicians to evaluate survival outcomes.

Several limitations of our study must be identified. Firstly, this was a retrospective study with a small sample size, which might have induced potential bias. Secondly, patients received 1G *EGFR*-TKI heterogeneity treatment involving gefitinib, erlotinib, and icotinib, and selection bias was inevitable. Thirdly, given the limited sample number and the efficacy of these two *EGFR*-TKIs against various exon 18 mutation subtypes, we did not analyze the efficacy of another 2G *EGFR*-TKI, dacotinib, or the 3G *EGFR*-TKI osimertinib. Finally, this study lacks the incidence of T790M beyond 1G *EGFR*-TKI and 2G *EGFR*-TKI resistance due to the low rate of re-biopsy or insufficient tissue specimens.

In conclusion, our data indicate that the combination of 1G *EGFR*-TKIs with chemotherapy was associated with a good response rate and a promising PFS outcome for NSCLC patients with uncommon *EGFR* exon 18 mutations. 1G *EGFR*-TKIs in combination with chemotherapy might be a feasible first-line treatment option, like afatinib. In clinical practice, when patients cannot tolerate the toxicity of afatinib, clinicians might use 1G *EGFR*-TKI in combination with chemotherapy for treatment of uncommon *EGFR* exon 18 mutations. Although the small sample size of patients with *EGFR* exon 18 mutations is a limitation, this trend in PFS that we found provides clues for further research and treatment. Future studies should determine the most appropriate treatment recommendation for NSCLC patients harboring uncommon *EGFR* exon 18 mutations.

## Data Availability Statement

The original contributions presented in the study are included in the article/**Supplementary Material**. Further inquiries can be directed to the corresponding author.

## Ethics Statement

The studies involving human participants were reviewed and approved by the institutional Ethics Review Board of National Cancer Center/Cancer Hospital, Chinese Academy of Medical Sciences and Peking Union Medical College (approval 18-070/1648). Written informed consent for participation was not required for this study in accordance with the national legislation and the institutional requirements.

## Author Contributions

HX and GY have contributed equally to this work and share first authorship. All authors have no conflicts of interest to declare.

## Funding

Beijing Hope Run Special Fund of Cancer Foundation of China (LC2019A15) & WUJIEPING Medical Foundation (320.6750.2020-05-4) and New National Natural Science Foundation (82072590).

## Conflict of Interest

The authors declare that the research was conducted in the absence of any commercial or financial relationships that could be construed as a potential conflict of interest.

## Publisher’s Note

All claims expressed in this article are solely those of the authors and do not necessarily represent those of their affiliated organizations, or those of the publisher, the editors and the reviewers. Any product that may be evaluated in this article, or claim that may be made by its manufacturer, is not guaranteed or endorsed by the publisher.

## Supplementary Material

Supplementary Figure 1 The study flow chart.

Supplementary Figure 2 **(A)** Disease progressive sites among all *EGFR* exon 18 mutations **(B)** Intrathoracic metastases with different treatment modalities **(C)** Brain metastases with different treatment modalities. (*EGFR*, epidermal growth factor receptor).
