# Supplementary figures and images for: EGFR Exon 18 Mutations in Advanced Non-Small Cell Lung Cancer: A Real-World Study on Diverse Treatment Patterns and Clinical Outcomes
Source: Front Oncol. 2021 Sep 2;11:713483. doi: 10.3389/fonc.2021.713483 (PMC8445032; doi:10.3389/fonc.2021.713483)

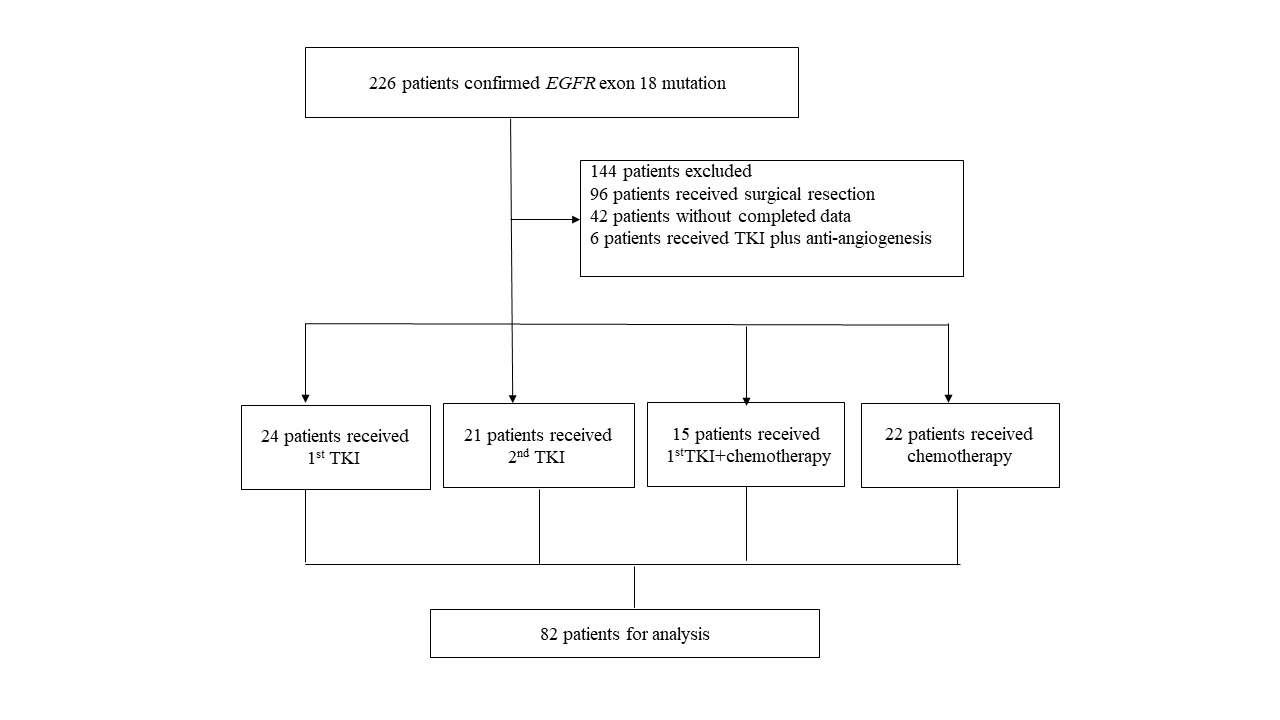

Supplement: Supplementary Figure 1 — The study flow chart. [file Image_1.tif]

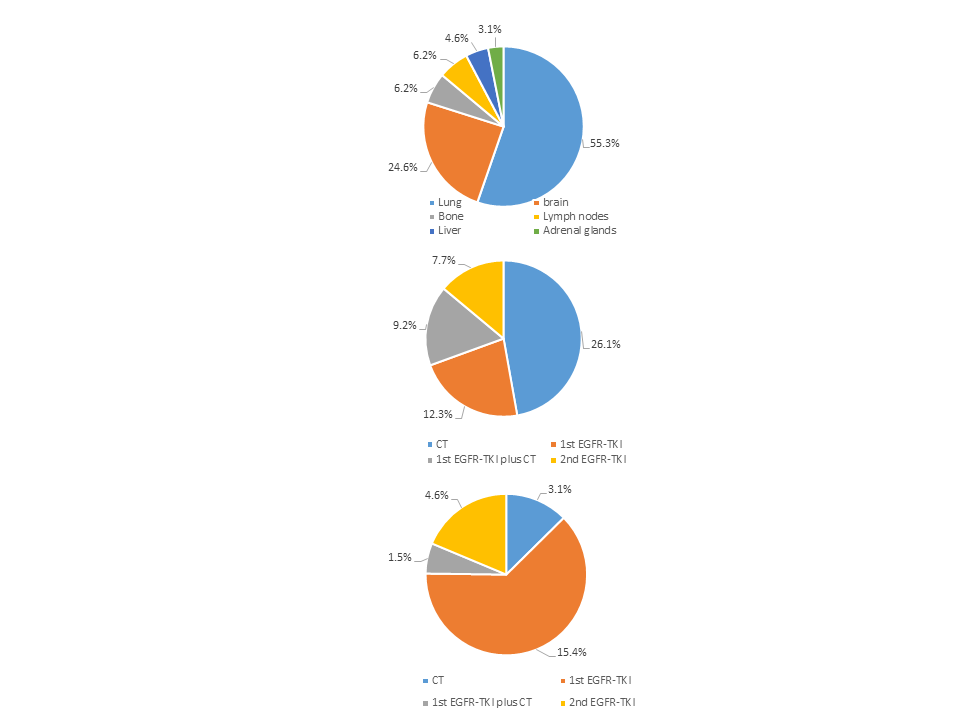

Supplement: Supplementary Figure 2 — (A) Disease progressive sites among all EGFR exon 18 mutations (B) Intrathoracic metastases with different treatment modalities (C) Brain metastases with different treatment modalities. (EGFR, epidermal growth factor receptor). [file Image_2.tif]
